# Intestinal deguelin drives resistance to acetaminophen-induced hepatotoxicity in female mice

**DOI:** 10.1080/19490976.2024.2404138

**Published:** 2024-09-21

**Authors:** Shenhai Gong, Yunong Zeng, Ze Wang, Yanru Li, Rong Wu, Lei Li, Hongbin Hu, Ping Qin, Zhichao Yu, Xintao Huang, Peiheng Guo, Hong Yang, Yi He, Zhibin Zhao, Weidong Xiao, Xiaoshan Zhao, Lei Gao, Shumin Cai, Zhenhua Zeng

**Affiliations:** a School of Traditional Chinese Medicine, Southern Medical University, Guangzhou, China; b Department of Critical Care Medicine, Nanfang Hospital, Southern Medical University, Guangzhou, China; c Department of Critical Care Medicine, The Third Affiliated Hospital of Southern Medical University, Guangzhou, China; d Henan Key Laboratory of Critical Care Medicine, Department of Critical Care Medicine and Department of Emergency Medicine, The First Affiliated Hospital of Zhengzhou University, Zhengzhou, China; e Department of Rheumatology and Immunology, The Third Affiliated Hospital, Southern Medical University, Guangzhou, China; f Medical Research Institute, Guangdong Provincial People’s Hospital, Southern Medical University, Guangzhou, China; g Department of General Surgery, Xinqiao Hospital, Army Medical University, Chongqing, China

**Keywords:** DILI, gut microbiota, gender variability, deguelin

## Abstract

Acetaminophen (APAP) overdose is a leading cause of drug-induced liver injury (DILI), with gender-specific differences in susceptibility. However, the mechanism underlying this phenomenon remains unclear. Our study reveals that the gender-specific differences in susceptibility to APAP-induced hepatotoxicity are due to differences in the gut microbiota. Through microbial multi-omics and cultivation, we observed increased gut microbiota-derived deguelin content in both women and female mice. Administration of deguelin was capable of alleviating hepatotoxicity in APAP-treated male mice, and this protective effect was associated with the inhibition of hepatocyte oxidative stress. Mechanistically, deguelin reduced the expression of thyrotropin receptor (TSHR) in hepatocytes with APAP treatment through direct interaction. Pharmacologic suppression of TSHR expression using ML224 significantly increased hepatic glutathione (GSH) in APAP-treated male mice. These findings suggest that gut microbiota-derived deguelin plays a crucial role in reducing APAP-induced hepatotoxicity in female mice, offering new insights into therapeutic strategies for DILI.

## 1. Introduction

APAP, a commonly used analgesic, is known to cause acute liver injury in a dose-dependent manner.^1,2^ Between 1998 and 2016, cases of DILI caused by APAP accounted for approximately 46% of all acute liver failure (ALF), with 28.5% of these cases resulting in death.^3^ Within the safe dosage range, APAP is mainly metabolized via glucuronidation and sulfation in the liver and subsequently excreted in the urine.^4–6^ However, when the intake of APAP exceeds the maximum metabolic capacity of the liver, the excess will be metabolized by cytochrome P450 enzymes (CYPs) into the cytotoxic-intermediate N-acetyl-p-benzoquinone imine (NAPQI). This intermediate is rapidly detoxified by binding with GSH.^7,8^ Once the hepatic GSH is completely consumed, unconjugated NAPQI covalently binds to cellular proteins to form APAP protein adducts.^9,10^ Our recent research demonstrated that gut microbiota-derived daidzein can prevent the accumulation of APAP protein adducts in the liver, thereby suppressing hepatocyte oxidative stress.^11^ Better understanding of the molecular mechanisms underpinning hepatocyte oxidative stress will advance our knowledge of the pathogenesis of APAP-induced hepatotoxicity.

Numerous preclinical studies have provided evidence supporting the existence of gender-specific variations in DILI, with females exhibiting higher resistance to drug toxicity compared to males.^12,13^ For instance, in a pyrrolizidine alkaloid-induced hepatotoxicity model, male mice displayed elevated serum levels of alanine aminotransferase (ALT) and aspartate aminotransferase (AST).^14^ Dai et al. reported that female C57BL/6 mice developed less severe liver injury after APAP overdose compared to their male counterparts.^15^ Although differences in the activity of CYP isozymes and the synthesis of GSH via glutamate-cysteine ligase may partly account for the gender-specific variability, the detailed molecular mechanisms underlying these differences are often unclear.^16,17^ A comprehensive investigation into the mechanisms responsible for the gender-specific differences in DILI will provide a basis for future clinical treatments.

The gut-liver axis has gained increasing recognition for its role in regulating the pathogenesis of DILI.^18,19^ Patients with DILI exhibit lower gut bacteria abundance and impaired bacterial metabolic pathways, particularly those related to carbohydrate and nucleoside biosynthesis.^20,21^ Exogenous supplementation of *Lactobacillus rhamnosus GG* has been demonstrated to prevent cholestatic DILI by activating the intestinal farnesoid X receptor-fibroblast growth factor 15 axis, consequently inhibiting hepatic bile acid synthesis.^22^ We previously demonstrated that the gut microbial metabolite 1-phenyl-1, 2-propanedione is responsible for APAP-induced rhythmic hepatotoxicity.^23^ This microbial metabolite depletes hepatic GSH and increases the production of APAP protein adducts. Furthermore, we recently found that the liberation of formononetin by β-galactosidase from *Parabacteroides merdae* mitigates sepsis-induced liver injury by preventing heterogeneous nuclear ribonucleoprotein U-like protein 2 mediated macrophage pyroptosis.^24^ The gut microbiota is in dynamic equilibrium and is often affected by gender.^25,26^ A study found that sex hormones were able to regulate the composition of the gut microbiota of 689 mice.^27^ Female mice exhibited higher abundances of bacterial genera, including *Dorea, Coprococcus and Ruminococcus*, compared to their male counterparts.^27^ Functionally, the gut microbiota of males can produce higher levels of trimethylamine and synthesize trimethylamine N-oxide, thereby contributing to an increased susceptibility to cardiovascular disease.^28,29^ Based on these findings, we hypothesized that the gut microbiota may be involved in the gender-related variability observed in APAP-induced hepatotoxicity.

The TSHR is a G-protein-coupled receptor located on the basolateral membrane of thyroid cells, with the function of controlling thyroid growth, division, and hormone secretion.^30,31^ The TSHR is also expressed in several other organs, including the skin, kidney, heart, liver, and skeletal muscle.^32–34^ In particular, hepatic TSHR regulates cholesterol synthesis by promoting 3-hydroxy-3-methyl-glutaryl coenzyme A reductase expression in a cyclic adenosine monophosphate/protein kinase A/cAMP-response element binding protein pathway-dependent manner, thereby increasing nonalcoholic fatty liver disease susceptibility.^35^ Additionally, the overexpression of TSHR exacerbates hepatic mitochondrial oxidative stress by enhancing cyclophilin D acetylation.^36^ Thus, hepatic TSHR may also be involved in the pathological processes associated with APAP-induced hepatotoxicity characterized by extensive oxidative stress.

In this study, we aimed to explore the influence of the gut microbiota on the gender-specific variation in APAP-induced hepatotoxicity. The study revealed that the gut microbiota metabolite deguelin can inhibit hepatocyte oxidative stress by downregulating hepatic TSHR expression and promoting resistance to APAP-induced hepatotoxicity in female mice.

## 2. Results

### 2.1. The enhanced resistance to APAP-induced hepatotoxicity in female mice is associated with gut microbiota

**Figure 1.**
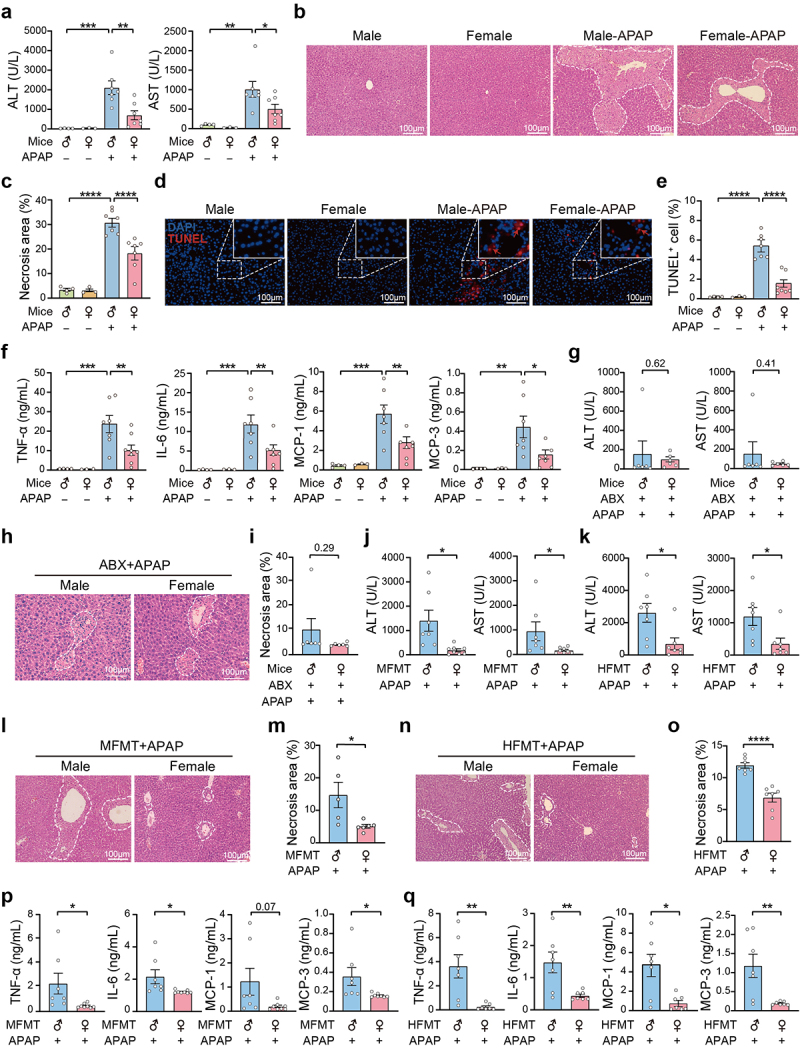
The enhanced resistance to APAP-induced hepatotoxicity in female mice is associated with gut microbiota.

To compare the resistance level of mice to DILI, we first established a stable APAP-induced liver injury model by grouping mice according to gender. As is shown in Figure 1(a), the serum levels of ALT and AST were much higher in male mice compared to their female counterparts after APAP overdose. With respect to pathologic features, extensive multifocal centrilobular hepatocellular death was observed in the liver of APAP-treated male mice compared to their female counterparts (Figures 1(b,c)). This observation was further confirmed through terminal deoxynucleotidyl transferase dUTP nick end labeling (TUNEL) staining (Figures 1(d,e)). Compared to the female mice, the concentrations of tumor necrosis factor-α (TNF-α), interleukin-6 (IL-6), monocyte chemoattractant protein-1 (MCP-1), and MCP-3 were increased in the blood of male mice after APAP exposure (Figure 1(f)). Next, both male and female mice were pretreated with a combination of antibiotics (ABX) to deplete the gut microbiota before being exposed to APAP (Figure S1a). Surprisingly, under these conditions, the serum concentrations of ALT and AST were indistinguishable between the treated male and female mice (Figure 1(g)). To further validate our findings, we quantified the percentage of hepatocyte death in APAP-treated male and female mice after depleting the gut microbiota (Figures 1(h,i) and S1b,c). Additionally, the fecal microbiota transplantation (FMT) experiment revealed that APAP-treated mice who had received feces from both female mice and women had lower serum ALT and AST levels (Figures 1(j,k)). This observation was substantiated by histopathological examination, which demonstrated that fecal transplantation from both female mice and women could mitigate the degree of multifocal centrilobular hepatocellular death in APAP-treated recipient male mice (Figures 1(l–o), S1d–g). Additionally, a significant decrease in inflammatory response was observed after APAP overdose in mice receiving both female mice and women feces (Figures 1(p,q)). On the contrary, fecal transplantation from male mice could exacerbate APAP hepatotoxicity in recipient female mice compared to those received feces from the female ones (Figures S1h–j). These findings indicate the gut microbiota is a key contributor to the resistance observed in female individuals against APAP-induced hepatotoxicity.

(a) Serum ALT and AST activities in male and female mice with or without APAP treatment for 24 h. n = 3–7. (b, c) Representative H&E staining images and quantification of necrotic areas in the liver of male and female mice with or without APAP treatment. n = 3–7. (d, e) Intrahepatic cell death evaluation using the TUNEL assay in male and female mice with or without APAP treatment. n= 3–7. (f) Quantification of serum proinflammatory cytokines and chemokines using ELISA in male and female mice with or without APAP treatment. n = 3–7. (g) Serum ALT and AST activities in male and female mice treated with APAP plus ABX. n = 6. (h, i) Representative H&E staining images and quantification of necrotic areas in the liver of male and female mice treated with APAP plus ABX. n = 6. (j, k) Serum ALT and AST activities in APAP-treated male mice after fecal microbiota transplantation from mice (MFMT) and humans (HFMT). n = 7–8. (i – o) Representative H&E staining images and quantification of necrotic areas in the liver of APAP-treated male mice after MFMT and HFMT. n = 5–7. (p, q) Quantification of serum proinflammatory cytokines and chemokines using ELISA in APAP-treated male mice after MFMT and HFMT. n = 7–8. Data were presented as mean ± SEM. Statistical analyses were performed using one-way ANOVA with Sidak post-hoc test (a – f) or two-tailed unpaired Student’s t-test (g – q). *p < 0.05, **p < 0.01, ***p < 0.001, and ****p < 0.0001. Scale bars: 100 μm.

### 2.2. Gut-derived deguelin is considerably abundant in female individuals

**Figure 2.**
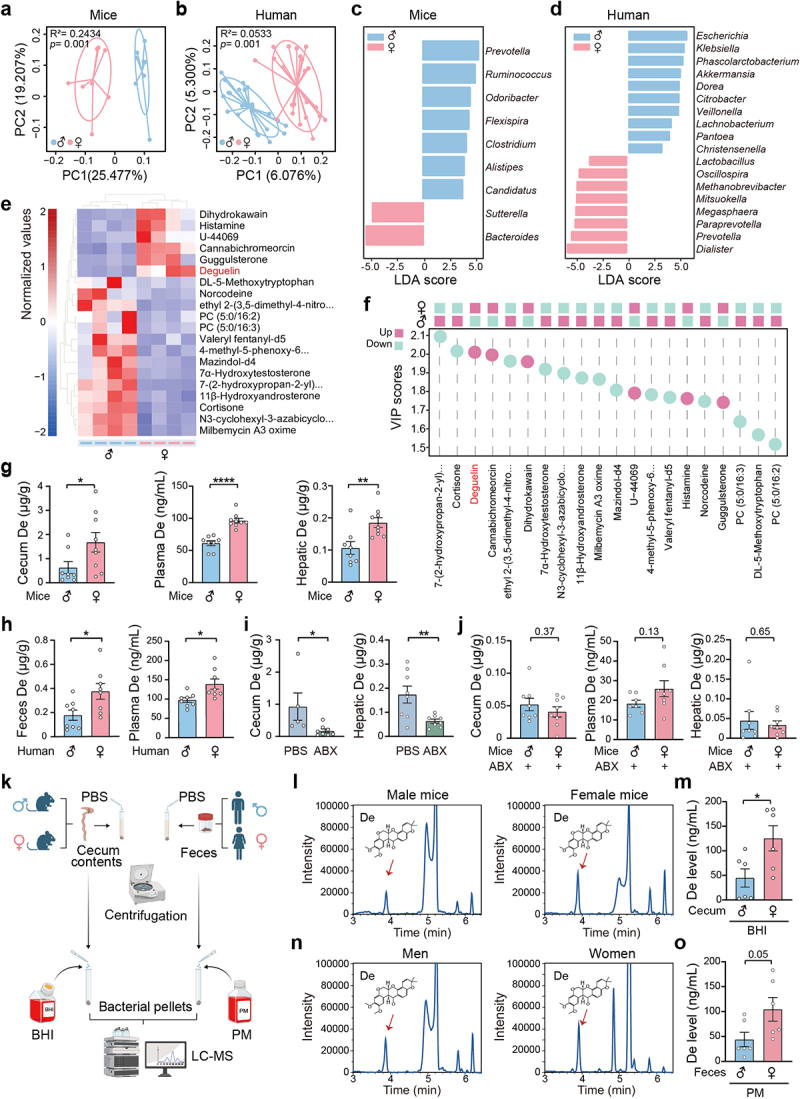
Gut-derived deguelin is considerably abundant in female individuals.

Subsequently, we explored how the gut microbiota contributes to the resistance observed in female individuals against APAP-induced hepatotoxicity. Principal coordinate analysis (PCoA) of 16 mouse fecal samples from 16S rRNA sequencing revealed significant differences in the distribution of gut microbiota between male and female mice (Figure 2(a)). A similar trend was observed in age-matched men and women (Figure 2(b)). At the phylum level, both female mice and women exhibited a higher *Firmicutes/Bacteroidetes* ratio compared to their male counterparts (Figures S2a,b). Specifically, the abundances of genuses such as *Bacteroides*, and *Sutterella* were higher in female mice than in male mice, whereas the abundances of *Dialister*, *Prevotella* and *Paraprevotella* (as the top three) were higher in women than in men (Figures 2(c,d)). In addition, we observed a prominent shift in the metabolic profiles of gut microbiota in female mice compared to their male counterparts (Figures S2c,d). Notably, the top 20 differential metabolites from cecal content of male and female mice, ranked by fold-change, were identified using a nontargeted metabolomic profiling approach (Figure 2(e)). The gut microbiota metabolites were then ranked based on their variable importance in projection (VIP) value. We found that among the top three metabolites based on VIP scores, only deguelin was elevated in female mice (Figure 2(f)). High-performance liquid chromatography (HPLC) analysis revealed that deguelin concentrations were higher in the cecal content, plasma, and liver of female mice compared to male mice (Figure 2(g)). These findings in mice were corroborated by observations in humans, as we also observed an increased level of deguelin in the feces and plasma of women compared to age-matched men (Figure 2(h)). To verify that cecal deguelin originates from the gut microbiota, mice were treated with ABX for 3 days. Deguelin concentrations in the cecal content and liver of the mice were significantly decreased after depleting the gut microbiota with ABX treatment (Figure 2(i)). Notably, the differences in deguelin concentrations between the female and male mice were diminished when ABX was administered (Figure 2(j)). Furthermore, we separately isolated fecal bacteria from female and male individuals and performed in vitro culture. Subsequently, we assessed the deguelin concentration in the bacterial supernatant using high-resolution LC-MS analysis (Figure 2(k)). As expected, the mixed fecal bacteria from both female mice and women were able to produce more deguelin in vitro (Figures 2(l–o)). We also found that specific bacterial strains, such as *Bacteroides stercoris*, abundant in female mice, displayed the ability to produce deguelin (Figures S2e,f). These findings prompted us to examine whether gut microbiota-derived deguelin is responsible for the resistance to APAP-induced hepatotoxicity in female mice.

(a) PCoA plot based on Bray-Curtis dissimilarity matrices of 16S rRNA sequence in female and male mice. n = 8. (b) PCoA plot based on Bray-Curtis dissimilarity matrices in women and men. n = 22. (c) Linear discriminant analysis effect size (LEfSe) revealed differences in bacterial profiles at the genus level between female and male mice. n = 8. (d) LEfSe showed differences in bacterial profiles at the genus level between men and women. n = 22. (e) Hierarchical clustering heatmap of the top 20 differential metabolites from cecal content of male and female mice ranked by fold change. n = 4. (f) The top 20 cecal metabolites were ranked using VIP scores. n = 4. (g) Quantification of deguelin in cecal content, blood, and liver of male and female mice using HPLC analysis. n = 8–9. (h) The concentrations of deguelin in feces and blood of women and men. n = 8. (i) The effect of ABX on the concentrations of deguelin in cecal content and liver of male mice. n = 5–8. (j) The concentrations of deguelin in cecal content, blood, and liver of male and female mice following ABX treatment for 3 days. n = 7–8. (k) Flowchart showing the experimental design of cecal bacterial mixtures of mice or fecal bacterial mixtures of human culture. Cecal content or feces was resuspended in PBS at a concentration of 0.125 g/mL, followed by gradient centrifugation. Fecal pellets were resuspended in brain heart infusion (BHI) or Postgate medium (PM) and anaerobically incubated for 24 h. Finally, bacterial culture supernatants were collected for the assessment of deguelin levels using high-resolution LC-MS analysis. (l, m) Deguelin concentration in culture supernatants of mouse cecal bacteria. n = 6. (n, o) Deguelin concentration in culture supernatants of human fecal bacteria. n = 6. Data were presented as mean ± SEM. Statistical analyses were performed using the Adonis test (a, b) and two-tailed unpaired Student’s t-test (g – o). Deguelin, De. *p < 0.05, **p < 0.01, ***p < 0.001, and ****p < 0.0001.

### 2.3. Deguelin treatment is effective in decreasing APAP-induced liver injury

**Figure 3.**
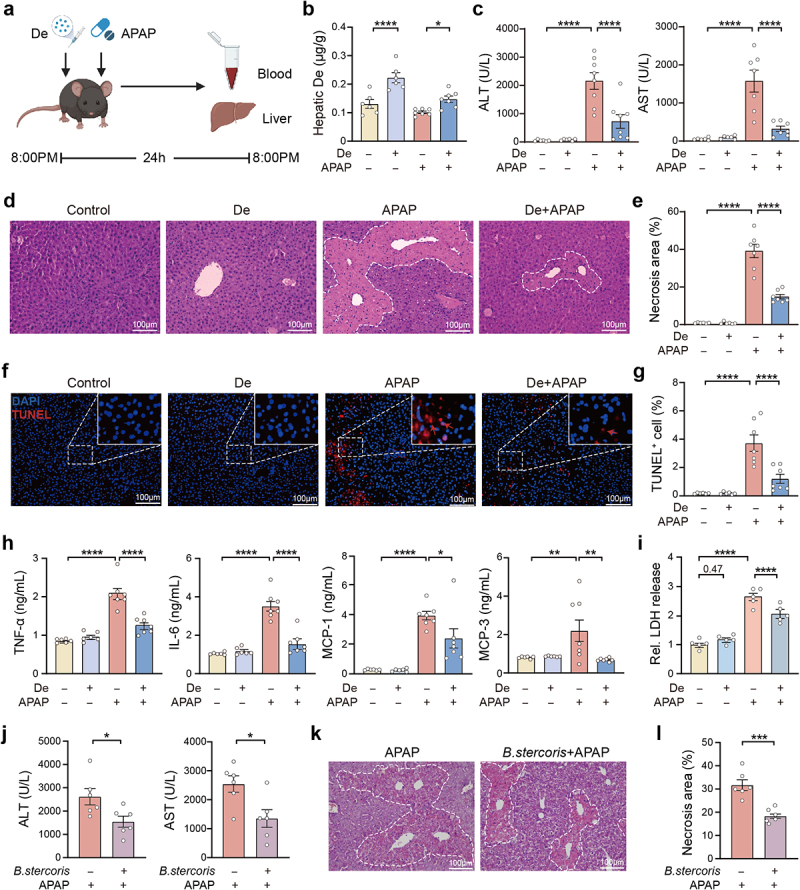
Deguelin treatment is effective in decreasing APAP-induced liver injury.

To validate our hypothesis, male mice were cotreated with APAP and deguelin, after which they were sacrificed and tissues were collected (Figure 3(a)). We first found that deguelin treatment alone did not affect body weight and cause any organ toxicity at therapeutic dose in mice (Figures S3a–h). Our observation revealed an accumulation of deguelin in the liver of the mice following deguelin treatment, whether administered in the presence or absence of APAP overdose, indicating that gut-derived deguelin can be taken up by the liver (Figure 3(b)). Deguelin reduced the degree of APAP-induced hepatotoxicity, as evidenced by a significant decrease in serum ALT and AST levels (Figure 3(c)). Moreover, deguelin treatment efficiently reduced the extensive multifocal centrilobular hepatocellular death caused by APAP overdose (Figures 3(d–g)). The concentrations of TNF-α, IL-6, MCP-1, and MCP-3 in the blood of APAP-treated mice declined significantly upon deguelin treatment (Figure 3(h)). We further assessed the protective effect of deguelin in vitro using primary mouse hepatocytes. Compared to vehicle-treated hepatocytes, deguelin treatment alone did not induce any cytotoxicity but exerted an attenuating effect on APAP-induced hepatocyte death, as indicated by lactate dehydrogenase (LDH) and cell counting kit-8 (CCK8) assays (Figures 3(i), S3i). We next wanted to explore whether *Bacteroides stercoris*, which is able to produce deguelin, protected against APAP-induced hepatotoxicity in mice. As expected, plasma ALT and AST levels were decreased in APAP-treated mice subjected to preadministration of *Bacteroides stercoris* (Figure 3(j)). Histopathological examination showed that mice subjected to *Bacteroides stercoris* pretreatment developed mild liver injury in the presence of APAP challenge (Figures 3(k,l)). Therefore, we are confident that gut-derived deguelin mediates the resistance to APAP-induced hepatotoxicity in female individuals.

(a) Schematic representation of the timeline of deguelin and APAP intervention. C57BL/6 male mice were treated intraperitoneally with 20 mg/kg deguelin or sesame oil, and then immediately given a single dose of 300 mg/kg APAP for 24 h via oral gavage. (b) Deguelin concentration in the liver of mice with or without deguelin administration in the presence or absence of APAP for 24 h. n = 6–7. (c) Serum ALT and AST activities in the mice treated as described above. n = 6–8. (d – g) H&E and TUNEL staining were performed to quantify necrotic areas and cell death percentages in the liver of the mice treated as described above. n = 6–7. (h) Quantification of serum proinflammatory cytokines and chemokines using ELISA in the mice treated as described above. n = 6–7. (i) Cell viability was determined using LDH release. The primary hepatocytes were pretreated with or without deguelin at a concentration of 0.1 μM for 2 h, followed by treatment with 5 mM APAP for 24 h. n = 5. (j) ALT and AST plasma activities in mice pretreated with *Bacteroides stercoris* (*B. stercoris*, 2 × 10^8^ CFU/mouse) once a day for 7 consecutive days, followed by administration of APAP for 24 h. n = 6. (k, l) Representative H&E staining images and quantification of necrotic areas in the liver of *B. stercoris* and APAP-treated mice. n = 6. Data were presented as mean ± SEM. Statistical analyses were performed using one-way ANOVA with Sidak post-hoc test (b – i) and two-tailed unpaired Student’s t-test (j – l). Deguelin, De; Relative, Rel. *p < 0.05, **p < 0.01, ***p < 0.001, and ****p < 0.0001. Scale bars: 100 μm.

### 2.4. Hepatic oxidative stress is suppressed by deguelin in APAP-treated mice

**Figure 4.**
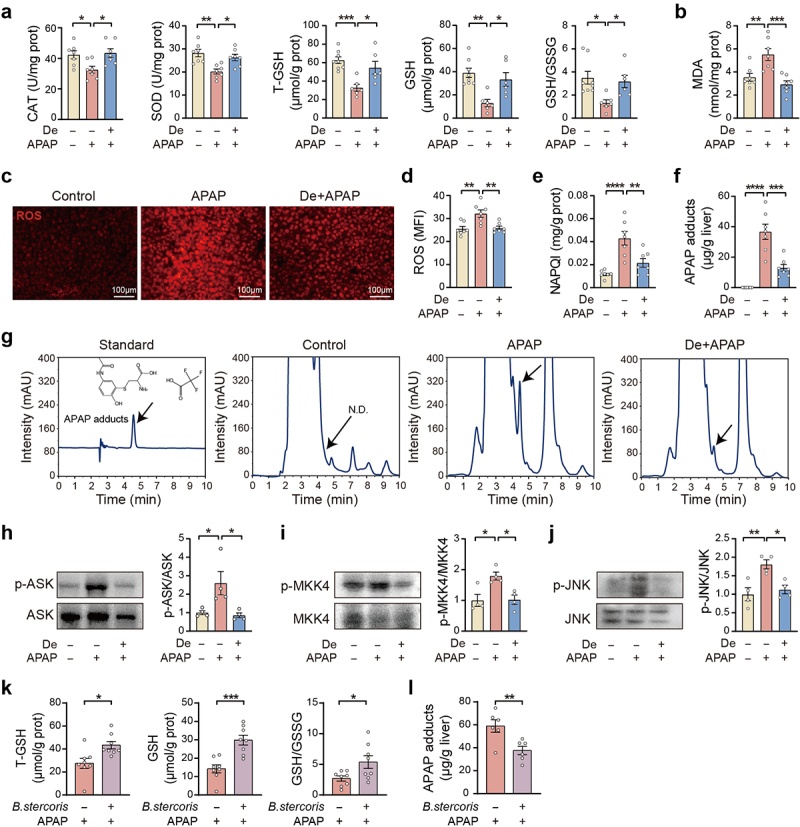
Hepatic oxidative stress is suppressed by deguelin in APAP-treated mice.

Given that oxidative stress in hepatocytes plays a central role in the pathophysiology of APAP-induced acute liver injury, we investigated the association between the hepatoprotective effect of deguelin and oxidative stress inhibition. We first found that urine APAP glucuronide (APAP-gluc) and APAP sulfate (APAP-sulf) concentrations were not altered by deguelin treatment in APAP-treated mice (Figure S4a). There were no differences in CYP2E1 and CYP1A2 protein levels between APAP group and APAP + deguelin group (Figures S4b, c). APAP excretion and hepatic CYP2E1 and CYP1A2 expressions were also indistinguishable between female and male mice (Figures S4d–g). In our mouse model, APAP treatment stably reduced the hepatic contents of antioxidant enzymes such as catalase (CAT), superoxide dismutase (SOD), and GSH, as well as the GSH/glutathione disulfide (GSSG) ratio, all of which were efficiently improved by deguelin treatment (Figure 4(a)). The levels of the lipid peroxide intermediate malondialdehyde (MDA), a marker of oxidative stress damage, decreased in the liver of the APAP-treated mice after deguelin administration (Figure 4(b)). We also found that deguelin reduced the accumulation of reactive oxygen species (ROS) in the liver after 1 h of APAP treatment (Figures 4(c,d)). Since APAP is primarily metabolized into the cytotoxic intermediate NAPQI and forms APAP protein adducts after GSH depletion, we examined the hepatic levels of these two products. There were significant decreases in hepatic levels of NAPQI and APAP protein adducts in the deguelin-treated mice after 1 h of APAP administration (Figures 4(e–g)). Additionally, deguelin significantly attenuated the enhanced phosphorylation of apoptosis signal-regulating kinase (ASK), mitogen-activated protein kinase 4 (MKK4), and c-Jun N-terminal kinase (JNK) in the liver of the APAP-treated mice (Figures 4(h–j)). In vitro, deguelin alleviated APAP-induced oxidative stress in primary hepatocytes, as evidenced by increased GSH levels and reduced ROS accumulation (Figures S4h–j). We also found that the level of hepatic oxidative stress in male mice higher than that in female mice upon APAP challenge (Figures S4k–m). Both fecal transplantation from female mice and *Bacteroides stercoris* administration were able to suppress hepatic oxidative stress in APAP-treated mice, as evidenced by decreased APAP protein adducts and higher GSH (Figures 4(k,l), and S4n, o). These findings support the conclusion that deguelin exerts a beneficial effect on APAP-induced liver injury by inhibiting hepatic oxidative stress.

(a, b) The hepatic levels of CAT, SOD, total GSH (T-GSH), reduced GSH, MDA, and GSH/GSSG ratio in vehicle or APAP-treated mice with or without deguelin administration. The liver samples were collected 1 h after APAP exposure. n = 6–8. (c, d) Intracellular ROS levels in frozen liver sections of the mice treated as described above. n = 7. (e – g) The hepatic concentrations of NAPQI and APAP protein adducts in the mice treated as described above. n = 7. (h – j) The expressions of p-ASK, p-MKK4, and p-JNK in the liver of mice treated as described above. n = 4. (k) The hepatic levels of T-GSH, reduced GSH, and GSH/GSSG ratio in vehicle or APAP-treated mice with or without *B. stercoris* administration. The liver samples were collected 1 h after APAP exposure. n = 8. (l) The hepatic concentration of APAP protein adducts in vehicle or APAP-treated mice with or without *B. stercoris* administration. The liver samples were collected 1 h after APAP exposure. n = 6. Data were presented as mean ± SEM. Statistical analyses were performed using one-way ANOVA with Sidak post-hoc test (a – j) and two-tailed unpaired Student’s t-test (k, l). Deguelin, De; *p < 0.05, **p < 0.01, ***p < 0.001, and ****p < 0.0001. Scale bars: 100 μm.

### 2.5. The inhibition of TSHR confers effect of deguelin against APAP-induced hepatocyte oxidative stress

**Figure 5.**
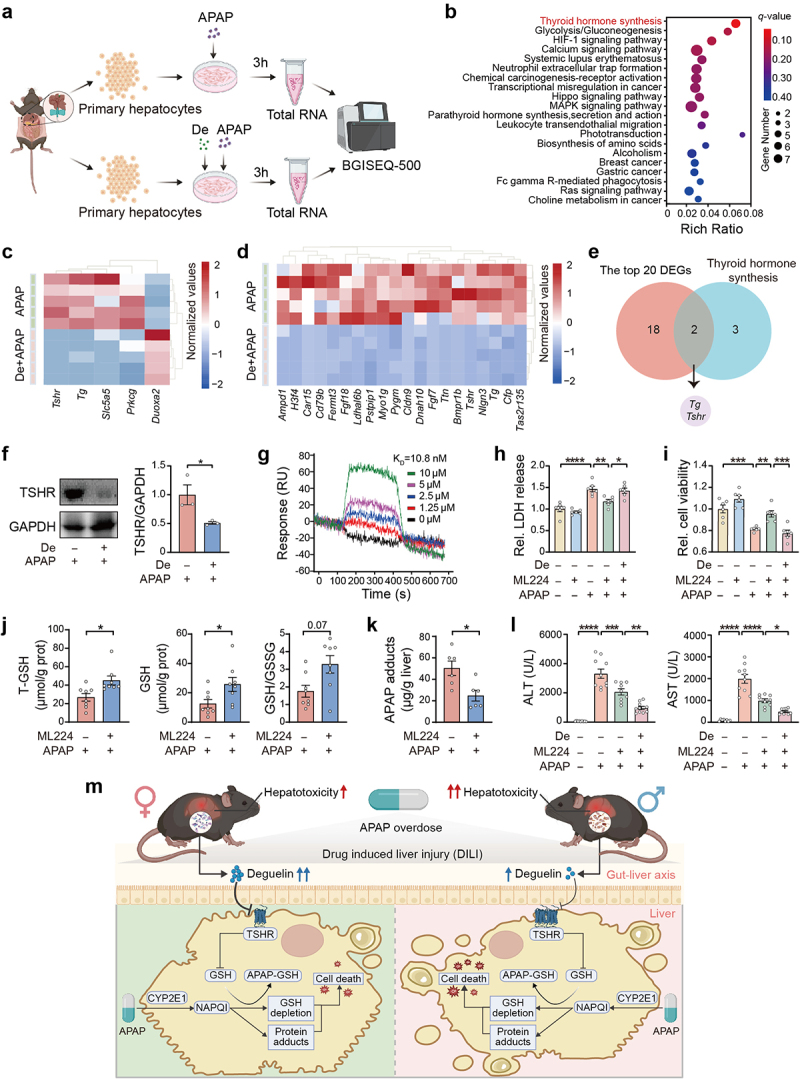
The inhibition of TSHR confers effect of deguelin against APAP-induced hepatocyte oxidative stress.

Up to now, the molecular target of deguelin in regulating APAP-induced hepatocyte oxidative stress remains unknown. To address this question, we conducted a detailed transcriptomic analysis of APAP-treated primary hepatocytes in the presence and absence of deguelin (Figure 5(a)). Compared to APAP alone, the administration of deguelin plus APAP produced completely different global gene expression profiles (Figure S5a). We then used a volcano plot to visualize the differentially expressed genes (DEGs) with fold changes greater than 4 between the two groups (Figure S5b). Subsequently, we performed a functional analysis of the 74 DEGs using the Kyoto Encyclopedia of Genes and Genomes (KEGG) database, and this analysis revealed that thyroid hormone synthesis pathway was most significantly altered by deguelin treatment (Figure 5(b)). Upon further investigation, we found that only 5 genes, including *Tshr*, *Duoxa2*, *Prkcg*, *Slc5a5*, and *Tg*, in the thyroid hormone synthesis pathway exhibited significant differences between the two groups (Figure 5(c)). In addition, a heatmap was generated to depict the top 20 DEGs ranked by their fold change differences in expression levels (Figure 5(d)). Venn diagrams showed an overlap of DEGs, including *Tshr* and *Tg*, between the thyroid hormone synthesis pathway and the top 20 genes selected by differential gene expression analysis (Figure 5(e)). A quantitative polymerase chain reaction (qPCR) analysis revealed a decrease in *Tshr* gene expression of primary hepatocytes and liver upon deguelin treatment in the presence of APAP overdose (Figure S5c). However, *Tg* was not consistently decreased in vitro and in vivo (Figure S5d). Therefore, we decided to focus on hepatic *Tshr* gene for further experimentation. Consistent with the mRNA expression, the protein level of TSHR was decreased in liver with deguelin treatment in the presence of APAP challenge for different time points (Figures 5(f), and S5e,f). Both fecal transplantation from female mice and *Bacteroides stercoris* administration decreased the hepatic expression of TSHR in APAP-treated mice (Figures S5g–j). To explore the molecular interactions between TSHR which is expressed predominantly on the surface of cell and deguelin, we performed a ligand-protein docking study and observed a direct interaction between deguelin and the predicted TSHR protein binding pocket (Figure S5k). Additionally, a surface plasmon resonance (SPR) analysis revealed that deguelin interacted directly with TSHR protein in vitro (Figure 5(g)). We then investigated the role of TSHR in APAP-induced hepatocyte oxidative stress. Notably, the inhibition of TSHR by ML224 alone did not cause cytotoxicity in primary hepatocytes (Figures 5(h,i)). At the same dose, ML224 treatment rescued the decrease in cell viability induced by APAP stimulation, but the protective effect was diminished when deguelin was used in combination with ML224, indicating that the protective effect of deguelin is associated with TSHR expression (Figures 5(h,i)). ML224 administration also suppressed APAP-induced hepatic oxidative stress in mice, as evidenced by an increase in GSH level, as well as a decrease in APAP protein adducts (Figures 5(j,k)). The suppressive effect of deguelin on hepatocyte oxidative stress upon APAP challenge was diminished when used in combination with ML224 (Figures S5l–o). Consistently, fecal transplantation from female individuals or *Bacteroides stercoris* administration also did not affect the level of hepatic oxidative stress in ML224-treated mice after APAP challenge (Figures S5p–s). We also observed the efficacy of ML224 in ameliorating APAP-induced liver injury in animal models (Figures 5(l), and S5t,u). To our surprise, the serum levels of transaminases and necrotic areas in the liver of APAP-treated mice were further reduced when deguelin was co-administered with ML224 (Figures 5(l),and S5t,u), indicating that the anti-oxidative property was partly responsible for the hepatoprotective effect of deguelin in the APAP-treated mice. In summary, we could conclude that deguelin improves APAP-induced hepatocyte oxidative stress through downregulation of TSHR.

(a) Flow chart outlining the preparation of primary hepatocytes for RNA sequencing. The primary hepatocytes were pretreated with or without 0.1 μM deguelin, followed by treatment with 5 mM APAP for 3 h. (b) KEGG pathway enrichment analysis of the 74 DEGs with fold change greater than 4. n = 5. (c) Heatmap showing the DEGs in the thyroid hormone synthesis pathway between the two groups. n = 5. (d) Heatmap showing the top 20 DEGs ranked by fold change differences in expression levels between the two groups. n = 5. (e) Venn diagram showing an overlap of DEGs between the thyroid hormone synthesis pathway and the top 20 DEGs. n = 5. (f) Relative protein level of TSHR in the liver of vehicle or APAP-treated mice with or without deguelin administration. The liver samples were collected 1 h after APAP exposure. n = 3. (g) SPR showed a direct interaction between deguelin and TSHR protein. The recombinant TSHR protein was immobilized on the SPRi chip. The deguelin flowed at increasing concentrations. (h, i) Cell viability was determined using LDH release and CCK8 assays. Primary hepatocytes were cotreated with or without 0.1 μM deguelin and 0.1 μM ML224, followed by treatment with 5 mM APAP for 24 h. n = 6. (j) The hepatic levels of T-GSH, reduced GSH, and GSH/GSSG ratio in vehicle or APAP-treated mice with or without 10 mg/kg ML224 cotreatment. The liver samples were collected 1 h after APAP exposure. n = 8. (k) The hepatic concentration of APAP protein adducts in vehicle or APAP-treated mice with or without ML224 cotreatment. The liver samples were collected 1 h after APAP exposure. n = 6. (l) Serum ALT and AST activities in mice cotreated with 20 mg/kg deguelin and 10 mg/kg ML224, immediately followed by APAP treatment for 24 h. n = 8–10. (m) Working model: The gut microbiota metabolite deguelin inhibit hepatocyte oxidative stress by downregulating TSHR expression, thereby mediating resistance to APAP-induced hepatotoxicity in female mice. Data were presented as mean ± SEM. Statistical analyses were performed using two-tailed unpaired Student’s t-test (f, j, k) and one-way ANOVA with Sidak post-hoc test (h, i, l). Deguelin, De; Relative, Rel. *p < 0.05, **p<0.01, ***p < 0.001, and ****p < 0.0001.

## 3. Discussion

APAP overdose is one of the major causes of acute liver injury, which is highly dynamic and heterogeneous.^13,23,37^ Numerous studies have noted a significant reduction in hepatotoxicity in mice when they are administered a single dose of APAP in the morning compared to nighttime administration.^23,38,39^ In addition, mice at child or adult ages were more sensitive to APAP-induced acute liver injury compared to infant mice.^40^ This dynamic variation in APAP-induced hepatotoxicity can be attributed to several possible factors, including (1) the differences in the expressions and activities of hepatic CYP450 enzymes, (2) the changes in rhythmic gene expression profiles, and (3) the decreased GSH pool size.^23,41,42^ However, the gut-liver axis provides new insights into the dynamic variation of APAP-induced hepatotoxicity. The gut microbiota influences liver diseases by translocating or secreting metabolites and outer membrane vesicles.^11,43–45^ Our previous study indicated that the gut microbiota-derived 1-phenyl-1, 2-propanedione is a key contributor to the diurnal variation of APAP-induced acute liver injury.^23^ In this study, the female mice exhibited a lower degree of hepatotoxicity compared to their male counterparts after APAP overdose (300 mg/kg). It has been documented that severe APAP overdose (600 mg/kg) obscures the difference in hepatotoxicity between male and female mice.^46^ We guessed that too high dose of APAP may thoroughly disrupt the difference in GSH synthesis among female and male mice. Additionally, most clinical studies demonstrated that acute liver injury induced by APAP overdose is more common and detrimental in women.^47,48^ The possible reason is that young females with intentional APAP overdoses accounted for most of the cases.^49^ Another possible contributing factor is the increased use of sedatives in female patients.^47^ The reason for the disparity of phenotype in humans and mice needs to be further explored. Next, given that there might be differences in the microbiome composition between female and male mice, we explored the mechanism underlying the gender-specific variability of APAP-induced hepatotoxicity from a gut perspective. To our excitement, the difference in hepatotoxicity between female and male mice upon APAP overdose was diminished after depleting the gut microbiota using ABX pretreatment. We further performed FMT experiments involving male and female individuals to validate the key role of gut microbiota in the gender-specific variability of APAP-induced hepatotoxicity. Notably, FMT from either female mice or women was able to alleviate APAP-induced acute liver injury in recipient male mice. We concluded that the gut microbiota is responsible for the resistance to APAP-induced hepatotoxicity in female individuals, shedding light on the innovative and clinically feasible strategy for DILI treatment.

We next sought to identify bacterial factors involved in the gender-specific variations in APAP-induced hepatotoxicity. Our 16S rRNA sequence analysis revealed a significant difference in the composition of gut microbiota between female and male mice and humans. Regrettably, we did not observe a consistent trend in the abundance of bacterial genera that was shared between female mice and women. However, the metabolomic and HPLC analysis indicated that the gut bacterial metabolite deguelin was enriched in both female mice and women. Consequently, we focused on the role of deguelin in the gender-specific variability of APAP-induced hepatotoxicity. Deguelin is a rotenoid natural product that possesses a wide range of functions, including inducing tumor apoptosis, inhibiting proliferation, and exerting anti-inflammatory effects.^50,51^ We found that deguelin was able to abrogate the APAP-induced increase of serum ALT and AST in the mice. The hepatoprotective effect of deguelin was directly associated with its antioxidant property. But the clinical applicability and safety of deguelin for DILI treatment need to be further studied.

In this study, we encountered an additional concern regarding the pathway by which gut microbiota-derived deguelin is generated. Although metabolome analysis revealed a higher level of deguelin in the gut of female individuals, we were uncertain whether deguelin generation was dependent on the gut microbiota. To address this question, we administered mice with oral ABX for 3 days to deplete the gut microbiota, after which we examined the fecal deguelin content. The experimental data suggested that deguelin is generated by the gut microbiota. However, the ABX experimental data did not completely address the concern regarding deguelin generation because ABX can also affect host metabolism.^52^ To get further evidence to support this argument, an in vitro bacterial culture experiment was performed. We found that mixed fecal bacteria from both female mice and women could generate more deguelin. In addition, we selected *Bacteroides stercoris* enriched in the gut of female mice for in vitro culture and similarly found that *Bacteroides stercoris* were able to directly generate deguelin. As is well known, there are two different mechanisms by which the gut microbiota generates metabolites. In the first mechanism, the whole genome of bacteria harbors genes involved in the direct synthesis of metabolites.^53^ In the second mechanism, the gut microbiota promotes the liberation of bioactive compounds from dietary food by encoding specific enzymes.^54^ Recently, Han et al. proposed the concept of celobiotics, in which *Bacteroides ovatus* releases N-methylserotonin from orange fiber through the action of specific enzymes, thereby reducing fat storage and improving glucose metabolism in the liver.^55^ In our laboratory, we previously found that the release of various plant-derived isoflavone aglycones, including formononetin and daidzein, depends on the action of bacterial β-galactosidase in the gut.^11,24^ Thus, these aglycones may be considered celobiotics. In this study, deguelin, as a phytochemical compound, is a naturally occurring rotenoid isolated from the roots of the genera *Lonchocarpus*, *Derris*, and *Tephrosia*.^56–58^ It is well known that natural compounds have poor bioavailability.^59^ Gut microbiota may promote the liberation of deguelin from plant-based foods through unknown enzymes, facilitating its absorption in the intestine. Therefore, we assumed that deguelin may act as a novel celobiotic. However, this interesting new hypothesis requires further studies.

One significant breakthrough in this study is the discovery of the pivotal role played by the TSHR in APAP-induced hepatocyte oxidative stress. The TSHR is primarily expressed in the basolateral membrane of thyroid cells and is involved in iodine uptake, thyroid hormone secretion, and thyroid follicular cell proliferation.^60,61^ It is also expressed in the liver and plays a crucial role in the pathogenesis of liver disease.^62^ For example, hepatic TSHR expression may be related to cholesterol synthesis and hepatic glucose production.^35^ The connection between hypothyroidism and nonalcoholic fatty liver disease has been well-documented, with one proposed explanation being that reduced thyroid hormone levels lead to decreased lipid utilization.^63–65^ Our experiment revealed that the expression of hepatic TSHR was decreased by deguelin through direct binding following APAP overdose. Additionally, the inhibition of TSHR expression by ML224 abolished the antioxidant effect of deguelin in APAP-treated mice. Prevention of APAP-induced hepatotoxicity was further enhanced when deguelin was co-administrated with ML224 in mice. We speculated that other cells, including macrophages and neutrophils, might play a role in the protective effects of deguelin against APAP-induced hepatotoxicity.

In summary, our study demonstrated that in female individuals, the gut microbiota can inhibit hepatocyte oxidative stress mediated by TSHR, primarily through the increased production of deguelin, leading to resistance against APAP-induced hepatotoxicity.

## 4. Materials and methods

### 4.1. Human samples

This study was conducted per the Declaration of Helsinki and approved by the Ethics Committee of Nanfang Hospital, Southern Medical University (NFEC-2023-306). Forty-four healthy adult volunteers (22 males and 22 females) were recruited from the Southern Hospital. All volunteers provided informed consent for the use of their fecal and blood samples in scientific research. The baseline characteristics for enrolled volunteers were listed in Table S1.

### 4.2. Animals and treatment

Eight-week-old male and female C57BL/6 mice were provided by SPF Biotechnology Co., Ltd. (Beijing, China). The experimental procedures were approved by the Animal Ethics Committee of Southern Medical University. The mice were housed in a controlled environment with 12 h light/dark cycles, a temperature of 25 ± 2°C, humidity between 40% between 60%, and had free access to water and food. Referring to our previous study,^11^ the ALF model was induced by APAP (300 mg/kg, Macklin, Shanghai, China) via oral gavage at 8:00 PM. To investigate the protective role of deguelin (dissolved in sesame oil) in ALF, the APAP-treated mice were immediately injected intraperitoneally with deguelin (20 mg/kg, InvivoChem, Guangzhou, China) or sesame oil for 24 h. For inhibitor experiment, ML224 (10 mg/kg, InvivoChem, Guangzhou, China) was injected intraperitoneally into mice.

### 4.3. Mouse primary hepatocyte isolation and cell culture

Mouse primary hepatocytes were isolated using collagenase type IV (Worthington, NJ, USA) following previously reported procedures.^66^ Briefly, mice were anesthetized, and liver was perfused with PBS and then digested with collagenase types IV at a flow rate of 6 mL/min via the portal vein. After the mash of liver, hepatocytes were resuspended in Roswell Park Memorial Institute (RPMI)-1640 medium (Gibco, CA, USA) supplemented with 10% fetal bovine serum (Gibco, CA, USA) and 100 U/mL penicillin/streptomycin (Gibco, CA, USA). Finally, mouse primary hepatocyte was seeded in collagen I (Corning, NY, USA)-precoated plates, followed by subjection to maintain in a suitable incubator at 37°C with 5% CO_2_. Mouse primary hepatocyte was used for experiments within 6 h of attachment.

### 4.4. Bacterial culture

*Bacteroides stercoris* was grown in a brain heart infusion (BHI) medium in an anaerobic environment at 37°C and subcultured every 24–48 h. *Bacteroides stercoris* was grown to a concentration of 10^9^ CFU/mL, and bacterial supernatant was used for HPLC detection. For the animal study, mice were gavaged with *Bacteroides stercoris* (2 × 10^8^ CFU/mouse) once a day for 7 consecutive days followed by APAP treatment for 24 h.

### 4.5. Fecal microbiota depletion and transplantation experiment

For depletion of the gut microbiota, ABX cocktail (200 mg/kg neomycin sulfate, 200 mg/kg metronidazole, 200 mg/kg ampicillin, and 100 mg/kg vancomycin) was orally administered to the mice once a day for 3 days. For the FMT experiment, feces from male and female individuals were suspended in sterile PBS at a concentration of 0.125 g/mL. Next, the fecal suspension was shaken and centrifuged at 1,000 rpm and 4°C for 5 min, after which the supernatant was collected. The supernatant was mixed with a volume of 20% sterile glycerol and stored at −80°C until transplantation. The recipient mice were pretreated with ABX for 5 days, and then received 0.2 mL fecal suspension from male or female individuals once a day for 3 days.^67^

### 4.6. In vivo fecal microbiota isolation and culture

The fecal contents were isolated from female and male mice and humans and then resuspended with PBS at a concentration of 0.125 g/mL. Fecal suspension was centrifuged at 1,000 g for 10 min, and 8,000 g for 5 min to obtain mixed bacterial pellets, followed by incubation in BHI medium or Postgate medium (PM) for 24 h under anaerobic conditions, respectively. Immediately after the incubation period, the level of deguelin in the bacterial culture supernatants was determined using high-resolution LC-MS analysis.

### 4.7. Biochemical and ELISA assays

Plasma ALT and AST activities were measured using commercial reagents (Nanjing Jiancheng Bioengineering Institute, Nanjing, China) as directed by the manufacturer. Commercial assay kits were used to measure GSH (Nanjing Jiancheng Bioengineering Institute, Nanjing, China), MDA (Nanjing Jiancheng Bioengineering Institute, Nanjing, China), SOD (Nanjing Jiancheng Bioengineering Institute, Nanjing, China), NAPQI (Boshen, Nanjing, China), CAT (Nanjing Jiancheng Bioengineering Institute, Nanjing, China), and GSSG (Nanjing Jiancheng Bioengineering Institute, Nanjing, China) in liver homogenates. The CytoTox 96® Non-Radioactive Cytotoxicity Assay Kit (Promega, WI, USA) and CCK8 (Meilunbio, Dalian, China) were separately used to detect LDH release and assess cell viability. Serum levels of TNF-α (Neobioscience, Shenzhen, China), IL-6 (Neobioscience, Shenzhen, China), MCP-1 (Neobioscience, Shenzhen, China), and MCP-3 (Cusabio, Wuhan, China) were measured using a corresponding commercial ELISA kit.

### 4.8. Histopathological analysis

Liver pathological changes were assessed using H&E staining. Intrahepatic cell death was evaluated using a TUNEL assay kit (KeyGEN, Nanjing, China). Random fields were captured using a microscope (Leica DMi8, Wetzlar, Germany), and ImageJ software was used to quantitatively evaluate the area of liver necrosis, the percentage of cell death as previously described.^11^

### 4.9. Quantitative reverse transcription PCR (qRT-PCR)

RNA was extracted using TRIzol reagent (Thermo Scientific, MA, USA) and retro-transcribed to cDNA using the ReverTra Ace qPCR RT Kit (Toyobo, Shanghai, China). The qPCR mixture consisted of 5 μL cDNA, 6 μL SYBR Green Master Mix (Toyobo, Osaka, Japan), and 1 μL specific primers. This mixture was assayed using a 7500 Real-Time PCR system (Applied Biosystems, Massachusetts, USA). The target gene expression level was normalized against the housekeeping gene 18S. The specific primer sequences are listed in Table S2.

### 4.10. Protein extraction and western blotting

The tissue homogenates were lysed using radioimmunoprecipitation assay lysis buffer (Thermo Scientific, MA, USA) containing protease or phosphatase inhibitors (Thermo Scientific, MA, USA). The protein concentration was determined using a BCA kit (Thermo Scientific, MA, USA). The proteins were separated using sodium dodecyl sulfate-polyacrylamide gel electrophoresis and transferred onto polyvinylidene difluoride membranes (Merck, MA, USA). The membranes were immunoblotted with primary antibodies, followed by secondary antibodies (Servicebio, Wuhan, China) for 1 h. The primary antibodies used were as follows: Phospho-ASK (Ser966) antibody (Affinity, Jiangsu, China), ASK antibody (Affinity, Jiangsu, China), MEK4/MKK4 antibody (Affinity, Jiangsu, China), Phospho-MEK4/MKK4 (Ser80) antibody (Affinity, Jiangsu, China), SAPK/JNK antibody (Cell Signaling Technology, MA, USA), phospho-SAPK/JNK (Thr183/Tyr185) antibody (Cell Signaling Technology, MA, USA), CYP2E1 (Proteintech, Wuhan, China), CYP1A2 (Proteintech, Wuhan, China), TSHR antibody (Affinity, Jiangsu, China) and GAPDH antibody (Proteintech, Wuhan, China). Enhanced chemiluminescence was conducted to visualize the protein bands. The expression levels of target proteins were quantitatively analyzed using ImageJ.

### 4.11. ROS levels

ROS production in liver was assessed using a commercial dihydroethidium (DHE) assay kit (Thermo Scientific, MA, USA), following the manufacturer’s instructions. Primary hepatocytes were co-incubated with a dichlorofluorescein diacetate (DCFH-DA) probe (Beyotime, Shanghai, China) for 20 min. Random fields were captured using a fluorescence microscope, and ImageJ was used for analysis.

### 4.12. Analysis of 16S rRNA sequencing

Fecal samples from female and male individuals were homogenized, and DNA was extracted using the DNA extraction kit (Mabio, Guangzhou, China) according to the manufacturer’s protocol. The obtained DNA sample was mixed with primers for the V4 region of bacterial 16S rRNA genes (forward primer: 5′-GTGTGYCAGCMGCCGCGGTAA-3′; reverse primer: 5′-CCGGACTACNVGGGTWTCTAAT-3′) and SYBR Green Master Mix for PCR amplification. The PCR products were analyzed using Illumina NovaSeq 6000 platforms. Data extraction, trimming, and peer-to-peer bioinformatic analysis were performed using QIIME2.

### 4.13. HPLC analysis

The levels of deguelin, APAP protein adducts, APAP sulfate (APAP-sulf) and APAP glucuronide (APAP-gluc) were assessed using HPLC (Agilent 1260, CA, USA). All samples were extracted with methanol and centrifuged at 15,000 g for 20 min. The supernatant was vacuum-dried, and the residue was redissolved in methanol before being injected into the HPLC. A sample volume of 10 μL was injected into a COSMOSIL 5C18-MS-II column (4.6 × 250 mm, 5 µm, Nacalai Tesque Inc., Kyoto, Japan). For the analysis of the APAP protein adducts, the mobile phase consisted of methanol and 0.1% (v/v) formic acid water at a volume of 20:80. For the determination of APAP-sulf and APAP-gluc, the mobile phase was 0.1% acetic acid acetonitrile and 0.1% acetic acid water at a volume ratio of 10:90. For the determination of deguelin, the mobile phase was methanol and water at a volume ratio of 70:30. The column temperature was maintained at 37°C, and the flow rate was 1.0 mL/min. Data were collected and analyzed using the Agilent LC1260 software.

### 4.14. Transcriptomic analysis

TRIzol reagent was used to extract RNA from primary hepatocytes. Library construction and Illumina sequencing were outsourced to BGI Co., Ltd. (Shenzhen, China). We performed functional annotation of DEGs using the KEGG database. We next used the clusterProfiler R package to assess the statistical enrichment of DEGs in thyroid hormone synthesis pathways. The enrichment results were corrected using the Benjamini-Hochberg method and adjusted *p*-values below 0.05 were considered significant.

### 4.15. Metabolomic analysis

Chromatographic separation was performed using a Vanquish UHPLC (Thermo Fisher, Germany) equipped with a Hypesil Gold column (100 × 2.1 mm, 1.9 μm, Thermo Fisher, USA). The mobile phase for positive mode was 0.1% formic acid (A) and methanol (B). For the negative mode, the mobile phase consisted of 5 mM ammonium acetate (A) and methanol (B). The method was as follows: starting from 2% B for 1.5 min, then from 2% B to 100% B over 10.5 min and held constant for 2 min. This was then reduced to 2% B over 0.1 min and held constant for 2.9 min. The flow rate was 0.2 mL/min and column temperature was kept at 40°C. Mass spectrometry data were collected using a Q Exactive HF mass spectrometer (Thermo Fisher Scientific, USA). The instrument operated with a full scan range of 100–1,500 m/z. The spray voltage was set to 3.20 kV for both positive and negative modes. Additionally, the sheath gas flow rate was set to 40 arb and the auxiliary gas flow rate was 10 arb. The capillary temperature was maintained at 320°C. Data were analyzed with Compound Discoverer 3.1 (Thermo Scientific, MA, USA) in combination with BGI metabolome, mzCloud and ChemSpider database.

### 4.16. LC-MS analysis

All samples were extracted with methanol and centrifuged at 15,000 g for 20 min. The supernatant was vacuum-dried, and the residue was redissolved in methanol. Following sample preparation, 10 μL of the supernatants was injected into a Shim-pack GIST C18-AQ column (Shimadzu, Kyoto, Japan), and samples were analyzed using a Nexera LC-30A HPLC system (Shimadzu, Kyoto, Japan) coupled with a Shimadzu LCMS-8050 Triple Quadrupole Mass Spectrometer (Shimadzu, Kyoto, Japan). Mobile phase A consisted of 0.1% formic acid in water, while mobile phase B consisted of methanol. The samples were eluted using the following gradients: 10–100% B, 5 min; 100% B, 3 min; 10% B, 3 min. The MS analysis was conducted using both positive and negative electrospray ionization ion sources. Deguelin was identified by the combination of mass-to-charge ratio (m/z = 393.2) and retention time (RT = 3.9 min) relative to authentic standard and peak areas were extracted and integrated using LabSolutions Chromatographic Data System in TIC model (Shimadzu, Kyoto, Japan).

### 4.17. SPR experiment

SPR were performed on the PlexArray HT A100 (Plexera, USA). The protocols were described as previously.^24^ Briefly, recombinant mouse TSHR protein (bs-42044P, Bioss, China) was immobilized on a Biacore 3D Dextran chip, and then activation with equal amounts of 0.49 M 1-(3-Dimethylaminopropyl)-3-ethylcarbodiimide hydrochloride and 0.1 M N-hydroxy-succinimide. The deguelin (dissolved in 1‰ dimethyl sulfoxide) flowed at increasing concentrations (1.25, 2.5, 5, 10 µM) at 2 μL/s. Data collection was accomplished via Plexera Data Explorer and analyzed using BIA evaluation software version 4.1.

## 5. Statistical analysis

All experimental data were calculated by using two-tailed unpaired Student’s t-test or one-way ANOVA with Sidak post-hoc test. Other statistical methods of sequencing data were described in the figure legends. *p < 0.05, **p < 0.01, ***p < 0.001 and ****p < 0.0001.

## Supplementary Material

Supplemental Material

## Data Availability

The transcriptome data were deposited in the NCBI SRA database under accession code PRJNA1042616. The 16S sequence data were deposited in the NCBI SRA database under accession code PRJNA1040406. The metabolome data have been deposited in the China National GeneBank DataBase under accession code CNP0005009.
