# Supplementary material for: Intestinal deguelin drives resistance to acetaminophen-induced hepatotoxicity in female mice
Source: Gut Microbes. 2024 Sep 21;16(1):2404138. doi: 10.1080/19490976.2024.2404138 (PMC11418218; doi:10.1080/19490976.2024.2404138)
Supplement: Supplemental Material [file KGMI_A_2404138_SM3590.docx]

**Supplementary Information:**

**Intestinal deguelin drives resistance to acetaminophen-induced hepatotoxicity in female mice**

Shenhai Gong^a,h^, Yunong Zeng^b,h^, Ze Wang^c,h^, Yanru Li^b,h^, Rong Wu^a^, Lei Li^d^, Hongbin Hu^b^, Ping Qin^a^, Zhichao Yu^a^, Xintao Huang^a^, Peiheng Guo^a^, Hong Yang^c^, Yi He^e^, Zhibin Zhao^f^, Weidong Xiao^g^, Xiaoshan Zhao^a^, Lei Gao^a^*, Shumin Cai^b^*, Zhenhua Zeng^b^*

^a^School of Traditional Chinese Medicine, Southern Medical University, Guangzhou 510515, China

^b^Department of Critical Care Medicine, Nanfang Hospital, Southern Medical University, Guangzhou, 510515, China

^c^Department of Critical Care Medicine, The Third Affiliated Hospital of Southern Medical University, Guangzhou, 510665, China

^d^Henan Key Laboratory of Critical Care Medicine, Department of Critical Care Medicine and Department of Emergency Medicine, The First Affiliated Hospital of Zhengzhou University, Zhengzhou, 450052, China

^e^Department of Rheumatology and Immunology, The Third Affiliated Hospital, Southern Medical University, Guangzhou, 510665, China

^f^Medical Research Institute, Guangdong Provincial People's Hospital, Southern Medical University, Guangzhou, 510080, China

^g^Department of general surgery, Xinqiao Hospital, Army Medical University, Chongqing, 400037, China

^h^These authors contributed equally

***Corresponding authors:** Zhenhua Zeng (zhenhuazeng.2008@163.com), Shumin Cai (13751845166@163.com) and Lei Gao (raygaolei@smu.edu.cn).

**
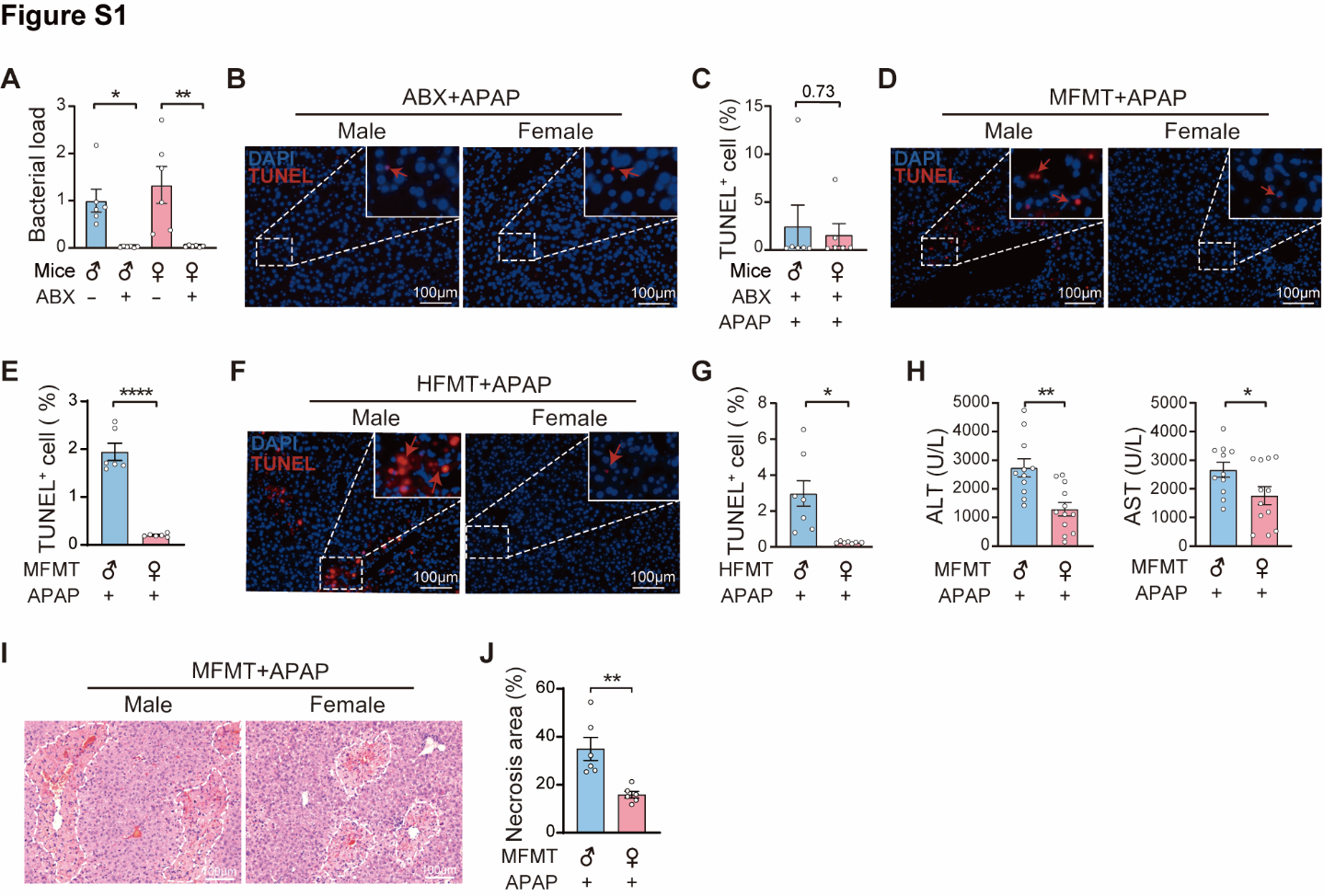
Figure S1. The enhanced resistance to APAP-induced hepatotoxicity in female mice is associated with gut microbiota.**

(A) Bacterial load in feces of male and female mice following ABX treatment for 3 days. n=6. (B, C) Intrahepatic cell death evaluation using the TUNEL assay in male and female mice treated with APAP plus ABX. n=6. (D, E) Intrahepatic cell death evaluation using the TUNEL assay in APAP-treated male mice after fecal microbiota transplantation from mice (MFMT). n=6. (F, G) Intrahepatic cell death evaluation using the TUNEL assay in APAP-treated male mice after fecal microbiota transplantation from human (HFMT). n=7. (H) Serum ALT and AST activities in APAP-treated female mice after MFMT. n=11–12. (I, J) Representative H&E staining images and quantification of necrotic areas in the liver of APAP-treated female mice after MFMT. n=6. Data were presented as mean ± SEM. Statistical analyses were performed using one-way ANOVA with Sidak post-hoc test (A) or two-tailed unpaired Student’s t-test (B–J). **p*<0.05, ***p*<0.01, ****p*<0.001 and *****p*<0.0001. Scale bars: 100 μm.


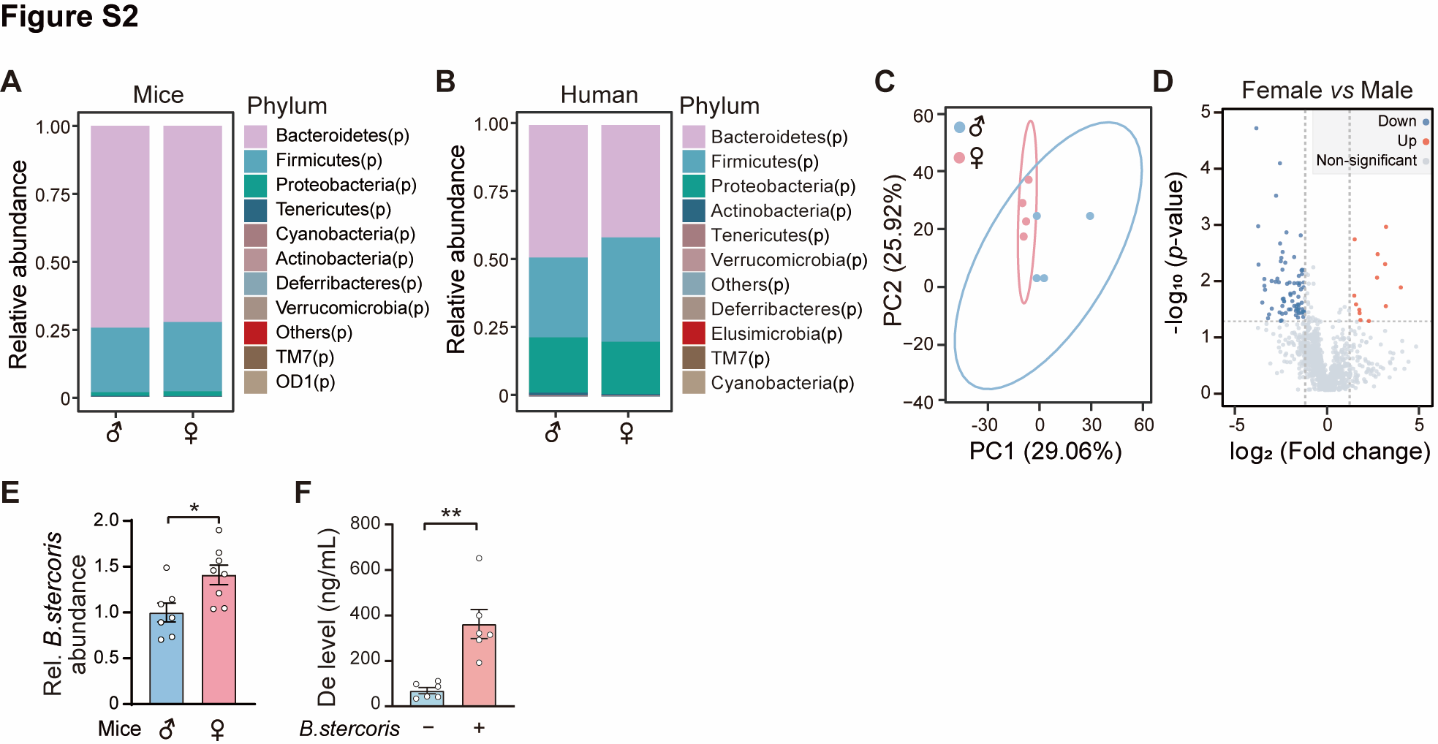


**Figure S2. Gut-derived deguelin is considerably abundant in female individuals.**

(A) Relative abundance of gut microbiota at the phylum level in female and male mice. n=8. (B) Relative abundance of gut microbiota at the phylum level in women and men. n=22. (C) Principal component analysis (PCA) for gut metabolites from female and male mice. n=4. (D) Volcano plot for gut metabolites from female and male mice. Data were compared by the Wilcoxon rank-sum test. n=4. (E) Relative abundance of *Bacteroides stercoris* (*B. stercoris*) in the gut of female and male mice. n=7–8. (F) Quantification of De using HPLC analysis in the bacterial supernatants of *B. stercoris*. BHI medium was used as a blank control. n=6. Data were presented as mean ± SEM. Statistical analyses were performed using two-tailed unpaired Student’s t-test (E, F). Deguelin, De; Relative, Rel. **p*<0.05, ***p*<0.01, ****p*<0.001, and *****p*<0.0001.

**
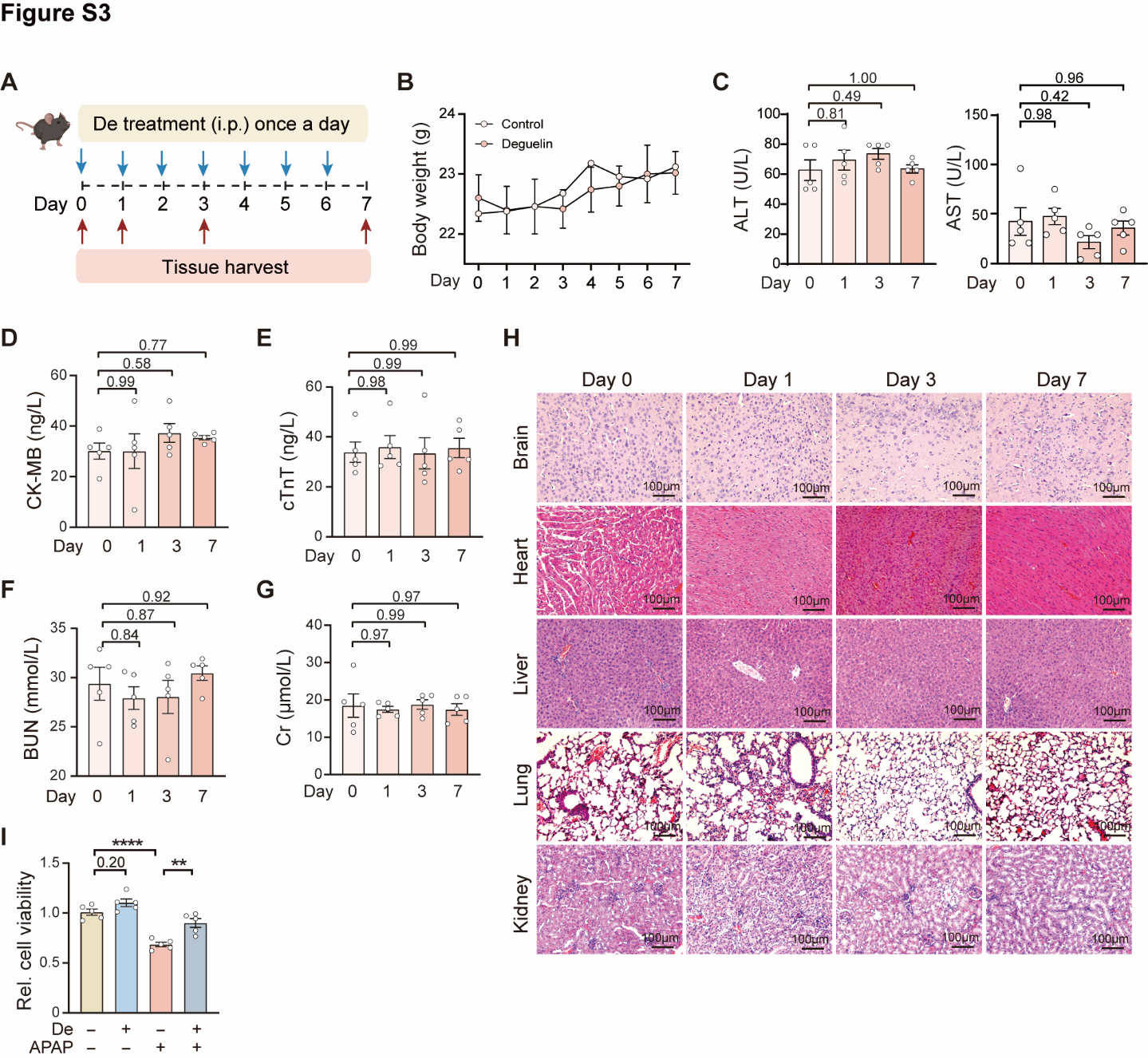
**

**Figure S3. The safety profile of deguelin treatment in mice.**

(A) Flow chart of experiment: mice were injected with 20 mg/kg deguelin for 7 consecutive days via an intraperitoneal route. Tissues were collected at different time points after APAP exposure. (B) Body weight was monitored during deguelin intervention. n=5. (C–G) The serum concentrations of ALT, AST, CK-MB, cTnT, BUN and Cr in mice during deguelin intervention. n=5. (H) Representative photomicrographs of H&E staining of brain, heart, liver, lung and kidney in mice during deguelin intervention. n=5. (I) Cell viability was determined using CCK8 assays. The primary hepatocytes were pretreated with or without deguelin at a concentration of 0.1 μM for 2 h, followed by treatment with 5 mM APAP for 24 h. n=5. Data were presented as mean ± SEM. Statistical analyses were performed using one-way ANOVA with Sidak post-hoc test. Deguelin, De; Relative, Rel. **p*<0.05, ***p*<0.01, ****p*<0.001, and *****p*<0.0001. Scale bars: 100 μm.

**
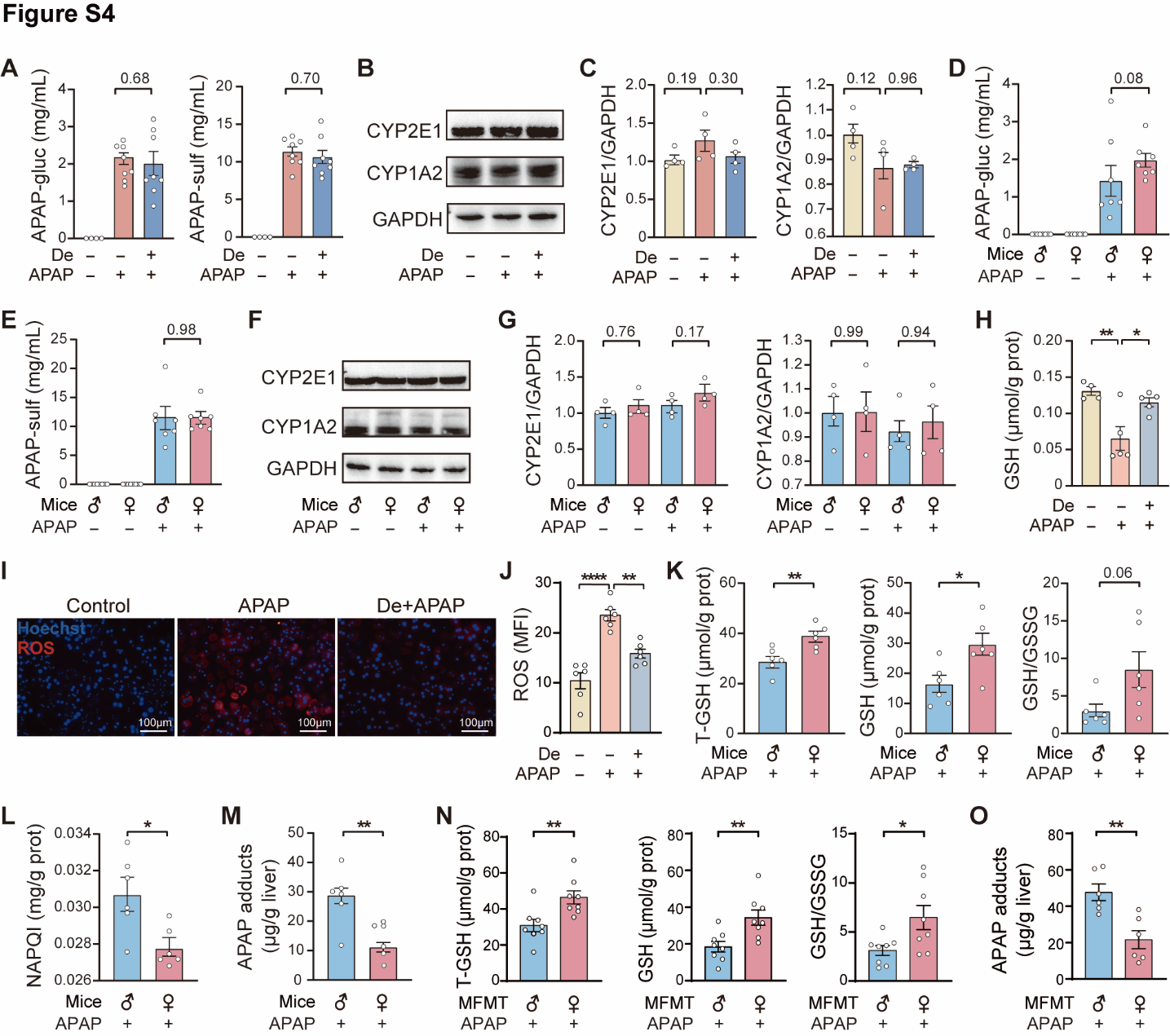
**

**Figure S4. Hepatic oxidative stress is suppressed by deguelin in APAP-treated mice**

(A) Measurement of APAP glucuronide (APAP-gluc) and APAP sulfate (APAP-sulf) concentrations in the urine of APAP-treated mice subjected to deguelin cotreatment for 1 h. n=4–8. (B, C) The hepatic expressions of CYP2E1 and CYP1A2 in APAP-treated mice subjected to deguelin cotreatment. Liver was collected at 1 h after APAP exposure. n=4. (D, E) Measurement of APAP-gluc and APAP-sulf concentrations in the urine of APAP-treated female and male mice. Urine was collected at 1 h after APAP exposure. n=7. (F, G) The hepatic expressions of CYP2E1 and CYP1A2 in male and female mice with or without APAP treatment for 1 h. n=4. (H) The levels of reduced GSH in primary hepatocytes pretreated with 0.1 μM deguelin for 2 h, followed by treatment with 5 mM APAP for 1 h. n=4–5. (I, J) The levels of ROS in primary hepatocytes pretreated with 0.1 μM deguelin for 2 h, followed by treatment with 5 mM APAP for 1 h. n=6–8. (K) Total GSH (T-GSH), reduced GSH and GSH/GSSG ratio in the liver of male and female mice with APAP treatment for 1 h. n=6. (L, M) The hepatic concentrations of NAPQI and APAP protein adducts in male and female mice with APAP treatment for 1 h. n=6. (N) T-GSH, reduced GSH and GSH/GSSG ratio in the liver of APAP-treated male mice after fecal microbiota transplantation from mice (MFMT). The liver samples were collected 1 h after APAP exposure. n=8. (O) The hepatic concentration of APAP protein adducts in APAP-treated male mice after MFMT. The liver samples were collected 1 h after APAP exposure. n=6. Data were presented as mean ± SEM. Statistical analyses were performed using one-way ANOVA with Sidak post-hoc test (A–J) or two-tailed unpaired Student’s t-test (K–O). Deguelin, De. **p*<0.05, ***p*<0.01, ****p*<0.001 and *****p*<0.0001. Scale bars: 100 μm.

**
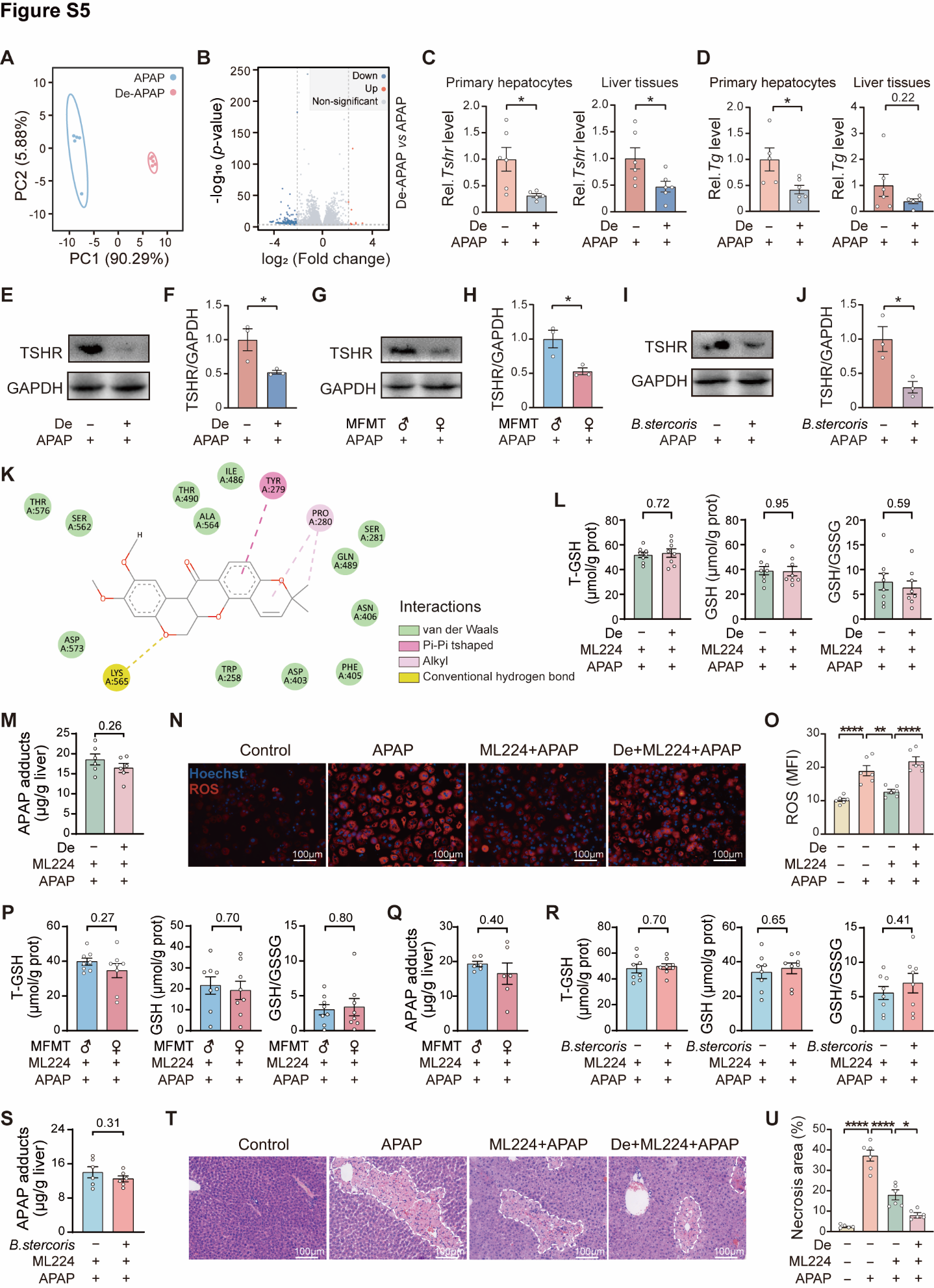
**

**Figure S5.** **The inhibition of TSHR confers effect of deguelin against APAP-induced hepatocyte oxidative stress.**

(A) PCA for gene profiles in the primary hepatocytes pretreated with or without 0.1 μM deguelin, followed by treatment with 5 mM APAP for 3 h. n=5. (B) Volcano plot displaying differentially expressed genes (DEGs) with fold change greater than 4 between the two groups. n=5. (C, D) Relative mRNA levels of *Tshr* and *Tg* in the liver of mice and primary hepatocytes. The primary hepatocytes were treated as described in (A). The mice were injected with or without 20 mg/kg deguelin in the presence of APAP for 24 h. n=5–6. (E, F) Relative protein level of TSHR in the liver of vehicle or APAP-treated mice with or without deguelin administration. The liver samples were collected 3 h after APAP exposure. n=3. (G, H) Relative protein level of TSHR in the liver of APAP-treated male mice after fecal microbiota transplantation from mice (MFMT). The liver samples were collected 1 h after APAP exposure. n=3. (I, J) Relative protein level of TSHR in the liver of vehicle or APAP-treated mice with or without *B. stercoris* administration. The liver samples were collected 1 h after APAP exposure. n=3. (K) A ligand-protein docking showing the direct interactions between deguelin and TSHR protein. (L, M) The concentrations of total GSH (T-GSH), reduced GSH, GSH/GSSG ratio, and APAP protein adducts in the liver of mice cotreated with 20 mg/kg deguelin and 10 mg/kg ML224, immediately followed by APAP treatment for 1 h. n=6–8. (N, O) Intracellular ROS level in the primary hepatocytes. The cells were cotreated with or without 0.1 μM deguelin and 0.1 μM ML224, followed by treatment with 5 mM APAP for 1 h. n=6. (P, Q) The concentrations of T-GSH, reduced GSH, GSH/GSSG ratio, and APAP protein adducts in the liver. Mice were subjected to MFMT, followed by APAP and ML224 cotreatment for 1 h. n=6–8. (R, S) The concentrations of T-GSH, reduced GSH, GSH/GSSG ratio, and APAP protein adducts in the liver. Mice were subjected to *Bacteroides stercoris* (*B. stercoris*) administration, followed by APAP and ML224 cotreatment for 1 h. n=6–8. (T, U) Representative H&E staining images and quantification of necrotic areas in the liver of mice cotreated with deguelin and ML224, immediately followed by APAP treatment for 24 h. n=5–6. Data were presented as mean ± SEM. Statistical analyses were performed using two-tailed unpaired Student’s t-test (C–M, P–S) or one-way ANOVA with Sidak post-hoc test (O, U). Deguelin, De; Relative, Rel. **p*<0.05, ***p*<0.01, ****p*<0.001 and *****p*<0.0001. Scale bars: 100 μm.

**Table S1. Baseline characteristics of volunteers**

|  | Men | Women |
| --- | --- | --- |
| **No. of cases (%)** | 22 (50%) | 22 (50%) |
| **Age (years)** |  |  |
| Median(range) | 21.5 (20.0-23.3) | 21.0 (20.0-24.0) |
| **Weight (kg)** |  |  |
| Median(range) | 63.6 (60.0-70.3) | 51.5 (48.0-55.0) |
| **Height (cm)**  Median (range) | 170.0 (168.0-175.0) | 162.5 (160.8-163.3) |

**Table S2. Sequence information for qPCR primers**

|  | **Forward primer (5’-3’)** | **Reverse primer (5’-3’)** |
| --- | --- | --- |
| ***18S*** | AGTCCCTGCCCTTTGTACACA | CGATCCGAGGGCCTCACTA |
| ***Tshr*** | TCATTGCCTCTGTAGACCTG | TGATAACTCACTGGCGAAA |
| ***Tg*** | TGTGGGTCTCTACTTTGTTGAGC | CGGAGTGGCTGTGCATCTAC |
| ***16S*** | GTGSTGCAYGGYTGTCGTCA | ACGTCRTCCMCACCTTCCTC |
| ***Bacteroides stercoris*** | GCTTGCTTTGATGGATGGC | CATGCGGGAAAACTATGCC |
